# Obesity and stroke: Can we translate from rodents to patients?

**DOI:** 10.1177/0271678X16670411

**Published:** 2016-09-21

**Authors:** Michael J Haley, Catherine B Lawrence

**Affiliations:** Faculty of Biology, Medicine and Health, University of Manchester, Manchester, UK

**Keywords:** Obesity, adiposity, stroke, inflammation, blood–brain barrier

## Abstract

Obesity is a risk factor for stroke and is consequently one of the most common co-morbidities found in patients. There is therefore an identified need to model co-morbidities preclinically to allow better translation from bench to bedside. In preclinical studies, both diet-induced and genetically obese rodents have worse stroke outcome, characterised by increased ischaemic damage and an altered inflammatory response. However, clinical studies have reported an ‘obesity paradox’ in stroke, characterised by reduced mortality and morbidity in obese patients. We discuss the potential reasons why the preclinical and clinical studies may not agree, and review the mechanisms identified in preclinical studies through which obesity may affects stroke outcome. We suggest inflammation plays a central role in this relationship, as obesity features increases in inflammatory mediators such as C-reactive protein and interleukin-6, and chronic inflammation has been linked to worse stroke risk and outcome.

## Obesity as a stroke co-morbidity

The detrimental effects of obesity on overall health are well understood, including an increased risk of developing hypertension and cardiovascular disease, and higher all-cause mortality.^1^ However, despite 35.5% of first-ever stroke patients being classified as clinically obese (EUROASPIRE III survey),^2^ we do not fully understand whether or how obesity affects stroke outcome. Stroke is already the leading cause of adult disability, accounting for 7% of the total disability-adjusted life years lost and 12% of total deaths in Europe.^3,4^ As most people affected by a stroke are over the age of 65, its prevalence and associated disabilities will likely increase due to the population living longer. As well as an ageing population, worldwide obesity has more than doubled since 1980, and in 2014, over 600 million adults were classified as obese (World Health Organisation (WHO) 2015).

Obesity is usually defined by body mass index (BMI, calculated as body weight in kg divided by square of height in m), with the WHO classifying adults with a BMI ≥ 25 kg/m^2^ as overweight and a BMI ≥ 30 kg/m^2^ as obese. Obesity can then further divided based on BMI into class 1 (30–34.9), class 2 (35–39.0) or class 3 (≥40) obesity. Several studies have identified that obesity is a risk factor for both ischaemic and haemorrhagic stroke in several ethnic populations and in both sexes.^5,6^ A linear relationship between increasing BMI and stroke risk has been reported for ischaemic stroke, though a J-shaped or no correlation is often reported for haemorrhagic stroke.^7–14^ Obesity is also a strong risk factor for the development of other risk factors for stroke including hypertension, diabetes and dyslipidaemia. However, even after adjustment for these potential co-founding factors, obesity has been found to be an independent risk factor for stroke.^5^

Currently, the only treatment for stroke is thrombolysis using tissue plasminogen activator (tPA) or by mechanical retrieval. However, since tPA can only be given up to 4.5 h post-stroke due to reduced efficacy and increased risk of haemorrhage, only a minority of stroke suffers benefit from tPA treatment.^15^ Furthermore, clinical evidence suggests that recanalisation of cerebral arteries after tPA therapy is reduced in obese stroke patients, suggesting a reduced efficacy of tPA.^16^ Similar resistance to thrombolysis has been reported in patients with the metabolic syndrome,^17,18^ of which obesity is a part. A potential explanation is that patients weighing over 100 kg may be underdosed, as the maximum recommended dose of tPA is 0.9 mg/kg (Guidelines for Management of Ischaemic Stroke and Transient Ischaemic Attack 2008).^19^ Indeed, worse outcome at 3 months in tPA-treated patients has been reported in patients who were >100 kg compared with patients who were <100 kg.^20,21^

**Figure 1.**
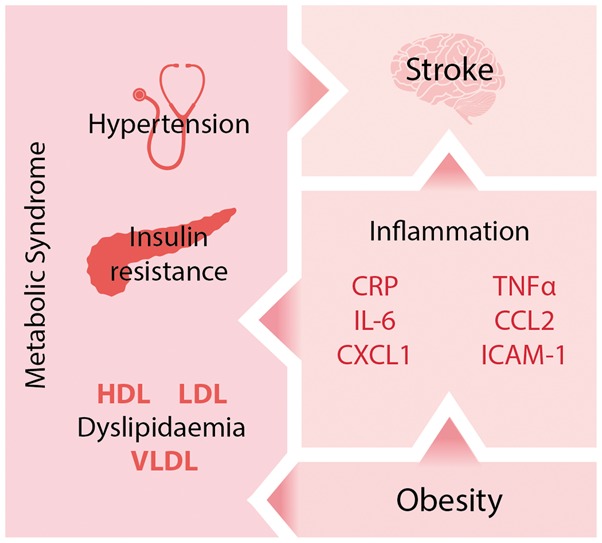
Obesity develops alongside other aspects of the metabolic syndrome and leads to chronic ‘low grade inflammation’. Systemic inflammation is known to affect stroke outcome, as are other aspects of the metabolic syndrome. CRP: C reactive protein; CCL2: chemokine (C-C motif) ligand 2; CXCL2: chemokine (C-X-C motif) ligand 2; ICAM-1: intracellular adhesion molecule 1; HDH: high-density lipoprotein; IL-6: interleukin-6; LDL: low-density lipoprotein; TNFα: tumour necrosis factor α; VLDL: very low-density lipoprotein.

In both obese and non-obese stroke patients, there is therefore an urgent need for new stroke treatments. Unfortunately, despite hundreds of stroke treatments showing efficacy in preclinical trials, most have been unsuccessful in the clinic. It is now believed that a key factor for this lack of translation from ‘bench to bedside’ is the failure of preclinical studies to consider fully the underlying status of a typical stroke patient. As age is the strongest risk factor for stroke, patients are usually elderly, and present with co-morbid diseases such as hypertension, vascular disease (e.g. atherosclerosis), diabetes, infection or metabolic syndrome/obesity. Several of these co-morbidities may co-exist in the same patient, and it is rare for a stroke to occur in an otherwise healthy individual (Figure 1).

## Obesity and chronic inflammation in stroke risk

Inflammation and immune mechanisms are well established factors in stroke risk and outcome, in both patients and animals.^22,23^ Obesity has classically been associated with disruption of pathways controlling lipid and glucose metabolism, however recent evidence has shown that obesity also has an inflammatory component (Figure 1). Measures of obesity, such as BMI, correlate with several markers of inflammation in patients. In particular, circulating concentrations of C-reactive protein (CRP) and interleukin (IL)-6 have been shown repeatedly to correlate positively with obesity^24–29^ in addition to tumour necrosis factor alpha (TNFα),^29^ monocyte chemoattractant protein 1 (MCP-1/CCL2),^30^ interleukin-8 (IL-8)^30^ and soluble intracellular adhesion molecule 1 (ICAM-1).^31^ This positive correlation between makers of inflammation and adiposity has led to suggestions that obesity is an inflammatory condition. However, the profile of immune activation found in obesity is quite different to that found in infection or injury, where the immune system is acutely activated to remove harmful stimuli. In obesity, the concentrations of proinflammatory markers are relatively low by comparison, but are maintained over long periods. Consequently, obesity has been described as resulting in “low-grade chronic inflammation”. This peripheral inflammatory response is thought to originate within adipose tissue, which becomes dysfunctional and inflamed during obesity.^32^ The important endocrine role of adipose tissue is highlighted by the growing family of adipose tissue-derived protein factors, collectively called adipokines (e.g. leptin and adiponectin). Besides having an established role in immune regulation and energy balance, adipokines have been found to regulate an expanding array of physiological functions, including haemostasis, lipid and glucose metabolism, blood pressure, insulin sensitivity and angiogenesis.^32,33^ The inflammation associated with obesity may lead to progressively increased stroke risk, since elevated concentrations of IL-6 and CRP in the plasma are both risk factors for stroke.^34–37^ In support of this, in obese and overweight patients, the BMI-associated cardiovascular disease (combined stroke and ischaemic heart disease) risk decreases substantially when adjusted for CRP, suggesting that inflammation is important in mediating the risk of stroke associated with obesity.^38^

## Stroke outcome in obese patients: The obesity paradox

Despite obesity being a risk factor for stroke, several studies have reported a protective effect of obesity on stroke outcome in patients. In these studies, patients are grouped by BMI on admission, and the presence of other stroke risk factors assessed. As would be expected, obese and overweight BMI groups have higher incidence of other risk factors including (but not limited to) hypertension, diabetes and dyslipidaemias. The relative impact of each factor on functional outcome is then assessed using multivariate statistics. When the contribution of other factors is adjusted for, higher BMI has been associated with reduced long-term all-cause mortality after stroke when compared with normal BMI groups in several populations.^39–47^ Furthermore, increased BMI has also been associated with other improved outcomes post-stroke, including a reduced risk of recurrent stroke,^44,48^ reduced morbidity,^42^ improved functional recovery^49^ and reduced short-term mortality.^40,48,50^

However, not all studies have confirmed the existence of an obesity paradox in stroke. For example, Ryu et al. found no association between increased BMI and long-term mortality after ischaemic stroke.^51^ Kim et al. found that higher BMI was associated with milder strokes on admission, and so when initial stroke severity was adjusted for there was no evidence of an obesity paradox in their cohort.^52^ In a study in patients receiving intravenous thrombolysis, body weight >100 kg was associated with increased mortality at 3 months post-stroke.^21^ The common use of long-term all-cause mortality as an end-point has also been criticised, for example when the Danish Stroke Register was reanalysed to only include deaths confirmed as occurring acutely due to the index stroke, there was no evidence of an obesity paradox.^53^ There is therefore still a lack of consensus about the obesity paradox in the literature; whether it predicts a currently unclear biological phenomenon, or is instead due to sampling or other methodological bias. A full discussion of these arguments is beyond the scope of this review, and have been previously discussed elsewhere.^54–56^ Overall, the clinical studies investigating stroke outcome in obese patients have focused on whether obesity has a positive or negative effect on long-term morbidity and mortality. However, the actual mechanisms through which obesity is proposed to have a beneficial or detrimental effect have undergone little to no investigation in clinical studies.

## Experimental stroke in obese rodents

In contrast with the clinical outlook, there is clear consensus in preclinical studies that obesity worsens stroke outcome. This detrimental effect of obesity on outcome has primarily been demonstrated in obese rodents undergoing experimental cerebral ischaemia. In both mouse and rat models of obesity, obese rodents suffer increased ischaemic brain damage and have worse behavioural outcomes in comparison with control animals (Table 1). This unfavourable effect was initially observed in genetic models of obesity, such as the *ob/ob* mouse,^57,63,78,80,84,86–88^ *db/db* mouse^74,75,77,91,92^ and Zucker rat,^76,79,83^ which all become obese due to a deficiency in the satiety hormone leptin or a defective leptin receptor. Since leptin usually acts on the hypothalamus to limit feeding, these animals (*ob/ob* and *db/db* mice, and Zucker rats) rapidly gain weight because they are hyperphagic. The appearance of ischaemic damage is also more rapid in *ob/ob* mice.^57^ Worse stroke outcome has also been confirmed in diet-induced models in which animals become obese due to being fed a high-fat diet. These detrimental effects of a high-fat diet have been observed in rat,^58,61,65,73,82,93^ mouse^60,64,66,88^ and gerbil^62,63,94^ models of diet-induced obesity. A variety of models of cerebral ischaemia have also been used in these studies, including transient and permanent occlusion of the middle cerebral artery, common carotid artery ligation and exposure to a low-oxygen environment. In mice, it has recently been shown that the negative impact of diet-induced obesity on stroke outcome in mice is dependent on how long the obese phenotype is present and the severity of the initial stroke insult (length of ischaemia).^60^ It is also unclear whether the detrimental effects of obesity on outcome in these preclinical studies are permanent, or reversible with weight loss.

**Table 1.** Summary of the studies that have reported outcome in obese animals, and the co-morbidities which they assessed.

Study	Species, strain	Obesity model	Ischaemia model	Effect of obesity on ischaemia outcome	Co-morbidities assessed				
					BW	Glu.	Ins.	Lip.	BP
Haley and Lawrence^57^	Mouse, *ob/ob*	Leptin receptor mutation	tMCAo	↑ Infarct volume	**↑**				
Cao et al.^58^	Rat, SD	8-week HFD	tMCAo	↑ Infarct volume	**↑**			**↑**	
Deng et al.^59^	Mouse, CD-1	10-week HFD (45%)	tMCAo	HFD-alone effect not assessed	**↑**	**↑**			
Maysami et al.^60^	Mouse, C57	3-/6-month HFD (60%)	tMCAo	↑ Infarct volume (6 months only on diet)	**↑**	**↑**			**↔**
Wu et al.^61^	Rat, SD	3-month Western diet	tMCAo	↑ Infarct volume					
Cheon et al.^62^	Gerbil	1-month HFD (60%)	Bilateral CCA	↑ Neuronal loss	**↔**	**↑**		**↑**	
Wu et al.^61^	Rat, SD	3-month Western diet	tMCAo	↑ Infarct volume					
Yan et al.^63^	Gerbil	1-month HFD (60%)	Bilateral CAA	↑ Neuronal loss	**↔**	**↑**		**↑**	
Deng et al.^64^	Mouse, C57	2.5-month HFD (45%)	tMCAo	↑ Infarct volume	**↑**	**↑**		**↑**	
Yang et al.^65^	Rat, SD	3-month HFD (45%)	tMCAo	↑ Neurological deficit	**↑**	**↔**	**↑**	**↑**	**↔**
Kim et al.^66^	Mouse, C57	2-month HFD (36%)	tMCAo	↑ Infarct volume	**↑**	**↑**	**↑**		
Herz et al.^67^	Mouse, ApoE	6-week Western diet	tMACAo	↑ Infarct volume					
Dhungana et al.^68^	Mouse, ApoE	15-week Western diet	pMCAo	**↔** Infarct or neurological deficit				**↑**	
Kim et al.^69^	Mouse, ApoE	11-week HFD (21%)	tMCAo	HFD-alone effect not assessed				**↑**	
Pradillo et al.^70^	Rat, *cp/cp*	Leptin receptor mutation	tMCAo	HFD-alone effect not assessed	**↑**				
Kawai et al.^71,72^	Rat, Zucker	Leptin receptor mutation	tMCAO	HFD-alone effect not assessed					**↑**
Langdon et al.^73^	Rat, SD	1- or 3-month Western diet	Endothelin injection	↑ Infarct volume (3 months only on diet)	**↑**				
Kumari et al.^74^	Mouse, *db/db*	Leptin receptor mutation	CCA ligation and hypoxia (8%)	↑ Infarct volume		**↑**			
Tureyen et al.^75^	Mouse, *db/db*	Leptin receptor mutation	tMCAo	↑ Infarct volume	**↑**	**↑**			
Ritter et al.^76^	Rat, Zucker	Leptin receptor mutation	tMCAo	↑ Infarct volume	**↑**	**↑**	**↑**		**↔**
Chen et al.^77^	Mouse, *db/db*	Leptin receptor mutation	tMCAo	↑ Infarct volume		**↑**			
Kumari et al.^78^	Mouse, *ob/ob*	Leptin mutation	CCA ligation and hypoxia (8%)	↑ Infarct volume		**↑**		**↑**	
Osmond et al.^79^	Rat, Zucker	Leptin receptor mutation	tMCAo	↑ Infarct volume	**↑**	**↑**	**↑**	**↑**	**↑**
McColl et al.^80^	Mouse, *ob/ob*	Leptin mutation	tMCAO	↑ Infarct volume	**↑**	**↔**			
Arvanitidis et al.^81^	Rat, SD	1-month Western diet (40%)	Forebrain ischaemia	**↔** Neuronal loss	**↑**	**↔**			**↔**
Deutsch et al.^82^	Rat, SD	10-week HFD (36%)	pMCAo	↑ Infarct volume	**↑**	**↑**	**↑**		**↑**
Osmond et al.^83^	Rat, Zucker	Leptin receptor mutation	tMCAo	↑ Infarct volume	**↑**	**↑**	**↑**	**↑**	**↑**
Valerio et al.^84^	Mouse, *ob/ob*	Leptin mutation	pMCAo	↑ Infarct volume					
Kim et al.^85^	Mouse, ApoE	2-month Western diet	tMCAo	↑ Infarct volume	**↑**			**↑**	
Terao et al.^86^	Mouse, *ob/ob*	Leptin mutation	tMCAo	↑ Infarct volume	**↑**	**↔**			**↔**
Mayanagi et al.^87^	Mouse, *ob/ob*	Leptin mutation	tMCAo	↑ Infarct volume		**↔**	**↑**	**↑**	**↑**
Nagai et al.^88^	Mouse, *ob/ob* & C57	15-week HFD (42%) for C57	pMCAo	↑ Infarct volume	**↑**	**↑**		**↑**	
Tureyen et al.^89^	Mouse, *db/db*	Leptin receptor mutation	tMCAo	↑ Infarct volume		**↑**			
Kumari et al.^90^	Mouse, *db/db*	Leptin receptor mutation	CCA ligation and hypoxia (8%)	*db/db*-alone effect not assessed					
Zhang et al.^91^	Mouse, *db/db*	Leptin receptor mutation	CCA ligation and hypoxia (8%)	*db/db*-alone effect not assessed	**↑**	**↑**			
Vannucci et al.^92^	Mouse, *db/db*	Leptin receptor mutation	CCA ligation and hypoxia (8%)	↑ Infarct volume	**↑**	**↑**			

An up arrow indicates an increase relative to non-co-morbid controls, a down arrow indicates a decrease, and double-headed arrow indicates no significant difference. A blank indicates the parameter was not reported. BW: body weight; BP: blood pressure; CCA: common carotid artery; Glu: blood glucose; HFD: high-fat diet (brackets indicate percentage of dietary calories which are fats); Ins: blood insulin; Lip: blood lipids; SD: Sprague Dawley; pMCAO: permanent MCAO; tMCAO: temporary MCAO.

Besides an increase in ischaemic damage, obese rodents often show an increase in blood–brain barrier (BBB) permeability and an increased incidence of haemorrhagic transformation,^57,60,61,64,74,77,80,86,88,93^ although the latter is not always observed in diet-induced rodents.^60^ This enhanced microvascular damage in obese animals likely contributes to their worse ischaemic damage due to the neurotoxic effects of both BBB breakdown and haemorrhagic transformation. Perhaps owing to the important role of the BBB in brain ion and water homoeostasis, an increase cerebral oedema is seen in obese rodents.^61,64,66,75,76,86,92^ White matter damage is also increased in obese rodents post-stroke.^77^ Importantly, cerebral oedema, BBB breakdown and haemorrhagic transformation are all indicators of poor prognosis in patients.^95–98^

## Effects of obesity on neuroinflammation in experimental stroke

The chronic low-grade inflammation resulting from obesity may affect stroke outcome by modulating central nervous system (CNS) inflammation prior to stroke. Obese patients often show increased levels of pro-inflammatory cytokines in the plasma, including CRP, IL-6, TNFα and CCL2/MCP-1.^28–30^ Circulating cytokines may access the brain parenchyma directly, or transduce the inflammatory signal over the BBB indirectly via receptors on the endothelial cells of the cerebrovasculature or by stimulation of afferent nerve fibres. However, the systemic elevation of inflammatory mediators in obesity is likely not great enough to cause an overt inflammatory response in the CNS. Instead, the low-level systemic inflammation found in obesity may prime cells to subsequent inflammatory stimuli, for example leukocytes and platelets in the circulation.^86,99^ Mice that become obese after being fed a high-fat diet show increased activation of astrocytes and microglia, and increases in inflammatory mediators in the brain, principally due to NF-κB activation.^100–104^ However, these responses could be due to dietary fats, rather than the resulting obesity. Increases in basal inflammation may therefore alter the reactivity of cells within the CNS to subsequent ischaemic stimuli, changing the subsequent inflammatory response.

Despite showing increased ischaemic damage, less microglial reactivity and reduced expression of inflammatory cytokines have been reported in the brains of both obese *db/db*, *ob/ob* and high-fat fed mice after cerebral ischemia.^66,78,86,90,91^ This may represent a failure to mount an appropriate inflammatory response, which may prevent the appropriate transition from acute inflammation to repair and recovery. Indeed, *db/db* mice show delayed and diminished expression of growth factor and other markers of repair in the brain after stroke.^90^ In contrast, other studies have shown cytokine and chemokine expression levels are increased in ischaemic brain tissue of obese rats and mice.^58,60,75,76^ This heightened central inflammatory response could be due to the increased damage in the obese rodents, although elevated expression of inflammatory mediators is observed as early as 2.5 h after reperfusion.^76^

## Effects of obesity on peripheral inflammatory response to experimental stroke

Stroke triggers a peripheral immune response by both humoural and neural routes, featuring lymphocyte release from immune organs (bone marrow, spleen and thymus), release of acute-phase proteins from the liver, increased inflammatory mediators in several organs, and sustained increases in other circulating markers of inflammation.^105–107^ Since this post-stroke peripheral response occurs as central damage is evolving, this process is thought to amplify the post-ischaemic inflammatory reaction and resulting damage. In obesity, the post-stroke peripheral immune response appears to be increased, as plasma IL-6, CCL2/MCP-1 and CXCL-1 (KC) significantly increase post-stroke in obese mice.^60,86^ Inhibition of CCL2/MCP-1 using a blocking antibody reduces the damage in *ob/ob* mice, suggesting the peripheral inflammatory response may contribute to damage in obesity.^86^ Similarly, both rosuvastatin (a statin) and darglitazone (a PPAR-γ agonist) were shown to be anti-inflammatory and neuroprotective in *ob/ob*, but not ob/− mice.^78,87^

## Obesity exacerbates microvascular disruption in experimental stroke

Evidence from rodent studies suggests that obesity exacerbates inflammatory disruption of cerebral microvessels, worsening stroke outcome. This is seen experimentally as an increase in BBB permeability, increased incidence of haemorrhagic transformation and worse cerebral oedema. This increase in BBB permeability in obese mice is seen within 4 h of reperfusion, where it appears to be mediated by an increase in transcytotic endothelial vesicles, rather than a loss of tight junction integrity.^57^ Vascular complications occurring later after reperfusion may be mediated by an upregulation of the matrix metalloproteinase (MMP) family of proteases. Increases in expression of both MMP-2 and MMP-9 have been found in the ischaemic hemispheres of obese rodents,^64,74,77,80,82^ and greater BBB breakdown, haemorrhagic transformation and ischaemic damage are absent in high-fat fed MMP-9 knock out mice^64^ suggesting a causative role of MMP-9 in the detrimental effects of obesity. MMP-9 is expressed by cerebral vessels after stroke in *ob/ob* mice,^80^ but MMP-9 may also originate from adherent leukocytes, particularly neutrophils that release large quantities of MMP-9 upon degranulation.^108^ In support of this, increased adhesion of neutrophils and other leukocytes are found in cerebral vessels of obese rodents post-stroke,^76,86^ as well as increased neutrophil recruitment in the parenchyma.^60,74^ Furthermore, the total number of circulating leukocytes is increased in obese people, including increases in monocyte and neutrophil abundance and oxidative burst activity^109–114^ and also in obese mice.^60,115^ Obese mice also show increased vascular inflammation after stroke, characterised by increased vascular ICAM-1 expression, which may mediate neutrophil recruitment.^58,75,76^ These data suggest that obesity exacerbates inflammatory processes converging at the brain microvasculature endothelium, resulting in leukocyte recruitment and BBB breakdown. However, it is currently unclear whether these effects are mediated by changes within or external to the vasculature. For example, obesity may result in changes to the vasculature that subsequently worsens the vascular response to stroke. Alternatively, they may be caused by the convergence of other non-vascular causes at the BBB.

Whether stroke leads to enhanced microvascular disruption in obese humans is much less clear. No study has yet compared BBB leakage in obese versus non-obese patients, or in patients with similar co-morbidities. However, in patients followed up within a week of ischaemic stroke, the risk of risk of haemorrhagic transformation was decreased in obese patients (BMI ≥ 25 kg/m^2^).^116^ Furthermore, in patients receiving intravenous thrombolysis after stroke, obesity had no effect on the development of haemorrhagic transformation.^117,118^ This is in contrast to genetically obese mice deficient in leptin (*ob/ob*) or its receptor (*db/db*), and diet-induced obese mice, which show an increased incidence of haemorrhagic transformation after experimental stroke.^57,64,77,80^

## Obesity affects cerebral vessel structure and tone

Obesity has effects on the structure and responsiveness of the cerebral vasculature that are likely to negatively affect stroke outcome. In high-fat fed and genetically obese rodents the middle cerebral arteries undergo remodelling that features a decrease in the lumen diameter and an increase in the thickness of the vascular wall, increasing vascular resistance.^64,82,83^ This structural remodelling seems dependent on the activity of MMPs 2 and 9, and features increased deposition of collagen 1 in the vascular wall.^64,82^ Obesity also affects the production of and response to factors that affect vascular tone. In particular, cerebral arteries from obese animals show increased vasoconstriction in response to 5-hydroxtryptamine (5-HT), potassium chloride (KCl) and endothelin-1 (ET-1), and reduced vasodilation in response to acetylcholine.^83,93,119^ However, other authors reported no effect of high-fat diet on vascular responsiveness, or attributed it to co-morbid hypertension.^79,82^ These changes may have a functional impact on cerebral blood flow, for example obese mice show an altered response in cerebral blood flow after whisker stimulation.^93,120^

Similar findings have also been made in the peripheral blood vessels of obese patients. Obesity is associated with increased intima-media thickness and decreased elasticity in arteries, impaired endothelium-mediated vasodilation and increased endothelin-1 activity.^121–124^ Although these observations have not been made directly in cerebral vessels, obese patients show other hallmarks indicative of cerebrovascular dysfunction. For example, increasing BMI is associated with lower cerebral blood flow velocity, increased cerebrovascular resistance and decreased cerebral blood flow.^125–127^ Therefore, both preclinical and clinical data suggest that cerebrovascular dysfunction characterised by increased vascular resistance and impaired autoregulation may contribute to worsened stroke outcome or risk in obesity.

## The relationship between altered adipokines in obesity and stroke

Plasma levels of adiponectin are high in healthy adults, but reduce in correlation with increasing adiposity.^128,129^ Adiponectin has a variety of anti-inflammatory actions, and has recently been shown to be neuroprotective in stroke.^130,131^ In humans, increased levels of plasma adiponectin correlate with decreased ischaemic stroke damage, and conversely, low levels are associated with increased mortality after stroke.^132^ Similar observations have been made in rodent models of transient focal ischaemia, with adiponectin administration decreasing infarct size and neurological deficit,^130,131^ and adiponectin-deficiency increasing damage.^133^ The neuroprotective effects of adiponectin appear to be primarily mediated via the ischaemic cerebrovascular endothelium where adiponectin selectively localises post-stroke. The mechanisms of this localisation are not fully understood since adiponectin is not synthesised locally and does not permeate an intact BBB,^134^ but may involve adhesion to collagens of the injured endothelium and passage over the ischaemically damaged endothelial barrier.^131,133^ The resulting accumulation of adiponectin may have a variety of beneficial effects. Adiponectin appears to promote BBB integrity by reducing BBB permeability, microvascular MMP-9 expression and parenchymal leukocyte accumulation.^131^ Furthermore, adiponectin induces endothelial nitric oxide synthase (eNOS) activation and consequentially increases cerebral blood flow during ischaemia.^130^ Finally, adiponectin has been shown to decrease expression of pro-inflammatory cytokines, purportedly by inhibiting NF-κB, possibly due to activation of adiponectin receptors.^131,134^ A similar decrease in inflammatory cytokine expression was found in cultured brain endothelial cells treated with adiponectin.^134^ Through these actions, adiponectin has been extensively shown to protect the vascular endothelium in the periphery.^135^ Therefore, a loss of these protective effects may explain increases in stroke damage and microvascular complications in obese mice where adiponectin levels are chronically decreased. In support, recent data demonstrate that obesity exacerbates experimental ischaemia by increasing apoptosis of adiponectin-expressing neurones.^61^

The adipokine leptin has also been investigated for its role in stroke outcome, with several authors reporting seemingly conflicting conclusions. Since leptin circulates at levels proportional to body fat, obesity results in an increase in plasma leptin concentration. However, because these high concentrations are sustained, obesity may result in desensitisation to central leptin signalling.^136^ Using lean mice, Zhang et al.^137^ reported that leptin dose-dependently reduced infarct volume and neurological deficits after transient focal ischaemia, suggesting leptin was neuroprotective. Furthermore, in leptin-deficient obese *ob/ob* mice administration of leptin was protective in permanent focal ischaemia^84^ but detrimental in transient focal ischaemia.^86^ In addition, a lack of any significant effect of leptin was reported in both lean and obese *ob/o*b mice.^80^ No conclusive explanation has been given for these conflicting reports, however differences in dose regimes, adiposity and reperfusion may be involved, suggesting that leptin’s role is more complicated than being merely pro- or anti-inflammatory.

## Obesity and other co-morbidities

Obesity is a component of the metabolic syndrome, and so is often accompanied by hyperglycaemia, hypertension and dyslipidaemia in patients that may all affect stroke outcome (Figure 1). Type-2 diabetes increases stroke risk and mortality,^138^ with acute hyperglycaemia resulting in greater infarcts and an increased risk of HT in rodents.^75,139^ Hypertension is the most important modifiable risk factor for stroke, and itself increases HT risk.^140^ Importantly, the pathological evolution of these conditions is intimately linked to obesity,^141^ so that disassociating the specific impact of obesity on health outcomes is difficult. This is the case in patients, but also in preclinical models of obesity that often develop different aspects of the metabolic syndrome dependent on their age, diet and genetic background (Table 1). For example, leptin-deficient *ob/ob* mice, *db/db* mice lacking the leptin receptor and mice fed a high fat diet all not only become obese, but eventually develop other aspects of the metabolic syndrome to some extent.^142,143^ Studies in obese rats have shown that presence of hypertension is critical in whether the detrimental effect of obesity on stroke outcome is observed.^79,83^ In contrast, the relationship between obesity and a worsened stroke outcome has been confirmed in mice without hypertension or hyperglycaemia.^60,80,86,93^ However, the lack of clinically relevant end-points (increased plasma glucose and blood pressure) does not mean their causative pathologies are not present and developing. This means that although animal models of obesity mimic the situation in obese people, the possible contribution from other co-morbidities should be considered when attempting to study specifically the effects of obesity on stroke. This is especially important when considering effects of obesity on the cerebral microvasculature where the deleterious effects on stroke outcome of both diabetes and hypertension are focused.^140,144^ However, similar considerations must be made for studies that have previously attributed poor outcome in animal models solely to the presence of diabetes or hypertension, when animals are often also obese.

## Reconciling preclinical and clinical outlooks

Despite the well-established link between obesity and stroke risk, several clinical studies have reported that obese and overweight stroke patients have improved mortality and morbidity. This is in stark contrast to the consensus of preclinical studies, which have clearly shown that obesity worsens stroke outcome in rodents. There are many potential reasons for this disparity, which will be discussed, including differences in experimental design, models, aims and outcomes between preclinical and clinical studies.

### Common limitations of preclinical experimental studies

The preclinical studies assessing the effects of obesity on stroke outcome are all experimental, and have several limitations in common. Whereas the clinical studies typically assess outcome in terms of functional recovery or mortality at weeks or years post-stroke, the majority of preclinical studies have focused on the evolution of the ischaemic lesion within 48 h of reperfusion. This focus on short-term outcomes may explain why no obesity paradox is found in preclinical studies. For example, a hypothesised reason that obesity is protective in stroke patients is that the metabolic reserves present in obesity protects against post-stroke weight loss, and associated muscle wasting.^145^ The metabolic consequences of stroke have been poorly studied pre-clinically in general, and even less so in the context of obesity. In preclinical studies, stroke is usually surgically induced in obese mice, rather than waiting for spontaneous strokes to occur. Therefore, if the obesity paradox is primarily due to the effects of obesity on risk, for example increasing the risk of a stroke earlier in life, then this would not be detectable in current animal studies. A further problem is that animals are almost all young and male. This tight control of variables, such as sex and age, is useful in establishing causal links in experimental studies, but does not reflect the clinical situation. A final disadvantage of preclinical studies is their exclusive use of rodents, and whether rodent physiology can accurately model human stroke. This question has been discussed in detail elsewhere.^146,147^

### Animal models of obesity and their co-morbidities

Worse stroke outcome has been reported in both genetically obese and diet-induced obese rodents, yet both models also develop other pathologies alongside their increased adiposity (Table 1). As discussed, the resulting phenotype in both models is not solely increased adiposity, but more similar to metabolic syndrome found in patients. This is an accurate recapitulation of the clinical situation, as individual patients are rarely solely obese, but often present with other co-morbidities, including hypertension, diabetes and dyslipidaemias.^40,42–44,46–48,50^ However, due to the presence of these other co-morbidities, it becomes difficult to attribute the worse outcome in rodent models as being solely due to obesity. This is in contrast to clinical studies that can quantify the specific contribution of obesity to stroke outcome by applying multivariate statistics to large, heterogeneous patient populations. By this reasoning, worse stroke outcome in obese rodents may not be due to their adiposity, but their development of dyslipidaemia, diabetes or vascular disease. However, the same may be true of the individual stroke patient, whose outcome will be determined by a constellation of common co-morbidities.

### Other considerations of obese models

Although both genetic and diet-induced obese models replicate the clinical situation found in obese patients, both have other drawbacks. Monogenic mutations leading to obesity are actually very rare in the general population and leptin has other immunological roles beyond regulation of energy balance.^148,149^ In diet-induced obesity, the constitution of the diet (principally the macronutrient ratio of fats, protein and carbohydrates) can affect the resulting phenotype. Use of an altered diet also adds an extra variable to consider, as effects on outcome may be due to the diet per se, rather the resulting obesity. Preclinical studies also use animals that are more homogenously and robustly obese than the patient population. Although this homogeneity makes interpretation of the contribution of obesity easier in experimental studies, it ignores the potentially protective effects of mild obesity (e.g. overweight). For example, mice fed a high-fat diet for 4 months show an average body weight increase of 60%.^60^ Assuming mice fed a control diet have a healthy BMI of 20–25, this translates to an increase in ‘mouse BMI’ to 32–40. In both mice and rats, the harmful effects of obesity on stroke outcome are not observed until a threshold level of obesity is obtained.^60,73^

### Common limitations of clinical observational studies

The clinical studies assessing the impact of obesity on stroke outcome are almost universally observational – experimental studies in this setting are not possible as they would require purposefully exposing patients to either stroke and obesity. For similar reasons, observational studies are limited in their potential outcomes as non-invasive techniques are preferred. Furthermore, very acute events after the onset of ischaemia are difficult to study clinically as patients may not arrive at hospital until hours after symptom onset, and even then their care must be prioritised. Therefore, such findings of faster evolution of damage in obese rodents would be difficult to replicate in patients.

A further potential problem with observational studies is their potential for selection bias. A typical epidemiological study assessing how obesity affects stroke outcome will include all patients in their cohort that present with ischaemic stroke on hospital admittance. However, stratifying based on the presence of ischaemic stroke introduces collider-stratification bias, a type of selection bias.^150,151^ This results in non-causal relationships or associations between effects (factors that can affect the risk of stroke), for example between obesity and age. For this reason, the average age of patients in the obese groups is lower than in the non-obese groups of several studies reporting on the obesity paradox, with age often decreasing inversely with BMI and reaching a difference of 10 years in the most obese groups.^42,47–51,53^ This problem is not insurmountable, as in the case of known and commonly measured effects, such as age and traditional cardiovascular risk factors, their impact can be adjusted for statistically. However, there are many potential factors that may affect stroke incidence that are not commonly measured, so-called unmeasured effects. Similarly to age, stratification based on the presence of stroke will also create non-causal relationship between these unmeasured effects and obesity due to collider-stratification bias. As we cannot statistically adjust or account for the presence of unmeasured effects, they can distort the relationship between obesity and stroke outcome. This confounding of the stroke-mortality relationship by unmeasured confounding effects has been hypothesised to account for all of,^151^ or at least part of,^152^ the obesity paradox.

The existing clinical studies have primarily used BMI as a measure of obesity, but BMI may not always be the best indicator of adiposity. This is primarily because BMI does not discriminate between lean body mass and fat mass, and obesity is associated with an increase in both lean and fat mass. Other measures of obesity may more accurately reflect harmful adiposity, for example visceral/abdominal obesity rather than BMI is more strongly correlated with metabolic health.^153,154^ In support of this, obesity has a beneficial effect on vascular disease survival in obese diabetic patients when obesity is classified by BMI, but not when classified by waist circumference.^155^ Furthermore, people with elevated BMI are not always obese (i.e. have increased adiposity) and conversely people within normal body weight by BMI can still show a high percentage of body fat that is associated with higher risk of disease.^156^ The usefulness of BMI as a measure of adiposity may also decrease with age. In a cohort of patients with chronic heart failure with mean age 62–66, BMI misclassified body fat status 41% of the time, and was a better indicator of lean body mass than adiposity.^157^

## Conclusion

Preclinical research has clearly shown that obesity worsens stroke outcome in rodents, suggesting a central role of pathological changes in the cerebrovasculature, including vascular inflammation and remodelling. In comparison, it is still unclear whether obesity worsens outcome in patients, and many preclinical findings cannot be replicated in the clinic. Future unification of preclinical and clinical findings will rely upon better alignment of research goals, with common outcomes that can be assessed in both fields. For example, studies assessing longer term functional outcomes in obese rodents could give insights into the obesity paradox in patients.

In this approach, preclinical studies should aim to make findings, which are verifiable in the clinic, and thereby give more confidence in translation potential of preclinical research.

**Figure 2.**
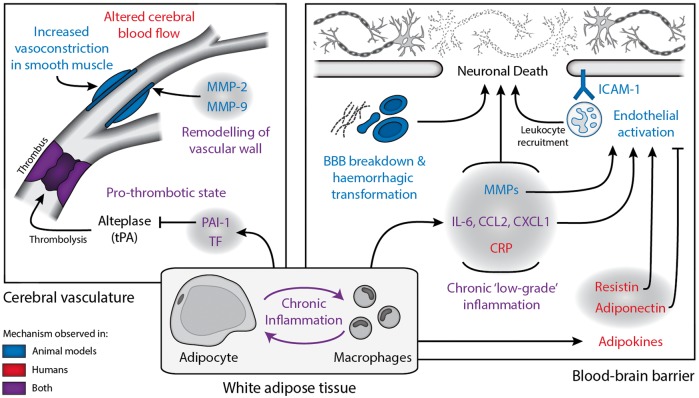
Summary of potential mechanism by which obesity may affect stroke outcome. The effects of obesity on stroke outcome appear to converge at the cerebral vasculature and the blood–brain barrier, with both animal and human studies suggesting that inflammation is key in mediating these effects. Mechanisms coloured in blue have been studied in obese animals undergoing experimental stroke, red coloured items have been observed in obese patients and purple in both obese animals and patients. This figure is not exhaustive, as there are many hypothetical ways in which obesity may affect stroke, which have not yet been studied. BBB: blood-brain barrier; CRP: C reactive protein; CCL2: chemokine (C-C motif) ligand 2; CXCL2: chemokine (C-X-C motif) ligand 2; ICAM-1: intracellular adhesion molecule 1; IL-6: interleukin-6; MMP: matrix metalloproteinase; PAI-1: plasminogen activator inhibitor 1; TF: tissue factor; tPA: tissue plasminogen activator.

Despite this disparity between the preclinical and clinical outlooks, several key mechanisms that may mediate the interaction between stroke and obesity have been identified (Figure 2), though the potential interactions not fully discussed here are extensive. This complexity is due to the wide-ranging effects of stroke and obesity on physiology, spanning cardiovascular, neurological, immunological and metabolic systems. Another complication for both preclinical and clinical research is that obesity commonly occurs with other conditions such as hypertension and diabetes.

At least in the preclinical data, there is strong evidence that altered inflammatory processes are central in mediating the worse stroke outcome found in obese rodents. Furthermore, there is a clinically established role of inflammation in both stroke and obesity. Further work is therefore required to reconcile the preclinical and clinical outlooks and allow identification of mechanisms important in both rodents and men, hopefully translating into better care for obese stroke patients.
